# Tumor Morphology for Prediction of Poor Responses Early in Neoadjuvant Chemotherapy for Breast Cancer: A Multicenter Retrospective Study

**DOI:** 10.3390/tomography10110134

**Published:** 2024-11-20

**Authors:** Wen Li, Nu N. Le, Rohan Nadkarni, Natsuko Onishi, Lisa J. Wilmes, Jessica E. Gibbs, Elissa R. Price, Bonnie N. Joe, Rita A. Mukhtar, Efstathios D. Gennatas, John Kornak, Mark Jesus M. Magbanua, Laura J. van’t Veer, Barbara LeStage, Laura J. Esserman, Nola M. Hylton

**Affiliations:** 1 Department of Radiology and Biomedical Imaging, University of California, 550 16th Street, San Francisco, CA 94158, USA; 2 Department of Surgery, University of California, San Francisco, 550 16th Street, San Francisco, CA 94158, USA; 3 Department of Epidemiology and Biostatistics, University of California, San Francisco, 550 16th Street, San Francisco, CA 94158, USA; 4 Department of Laboratory Medicine, University of California, San Francisco, 2340 Sutter Street, San Francisco, CA 94115, USA; 5 I-SPY 2 Advocacy Group, San Francisco, CA 94158, USA

**Keywords:** magnetic resonance imaging, breast cancer, tumor morphology, neoadjuvant therapy, multicenter clinical trial, residual cancer burden

## Abstract

Background: This multicenter and retrospective study investigated the additive value of tumor morphologic features derived from the functional tumor volume (FTV) tumor mask at pre-treatment (T0) and the early treatment time point (T1) in the prediction of pathologic outcomes for breast cancer patients undergoing neoadjuvant chemotherapy. Methods: A total of 910 patients enrolled in the multicenter I-SPY 2 trial were included. FTV and tumor morphologic features were calculated from the dynamic contrast-enhanced (DCE) MRI. A poor response was defined as a residual cancer burden (RCB) class III (RCB-III) at surgical excision. The area under the receiver operating characteristic curve (AUC) was used to evaluate the predictive performance. The analysis was performed in the full cohort and in individual sub-cohorts stratified by hormone receptor (HR) and human epidermal growth factor receptor 2 (HER2) status. Results: In the full cohort, the AUCs for the use of the FTV ratio and clinicopathologic data were 0.64 ± 0.03 (mean ± SD [standard deviation]). With morphologic features, the AUC increased significantly to 0.76 ± 0.04 (*p* < 0.001). The ratio of the surface area to volume ratio between T0 and T1 was found to be the most contributing feature. All top contributing features were from T1. An improvement was also observed in the HR+/HER2- and triple-negative sub-cohorts. The AUC increased significantly from 0.56 ± 0.05 to 0.70 ± 0.06 (*p* < 0.001) and from 0.65 ± 0.06 to 0.73 ± 0.06 (*p* < 0.001), respectively, when adding morphologic features. Conclusion: Tumor morphologic features can improve the prediction of RCB-III compared to using FTV only at the early treatment time point.

## 1. Introduction

For women with locally advanced breast cancer undergoing neoadjuvant chemotherapy (NAC), favorable recurrence-free survival (RFS) and overall survival (OS) are observed for those who achieve a pathologic complete response (pCR), defined as the absence of invasive disease after NAC [1,2]. For those who do not have pCR, the residual cancer burden (RCB) method is a standard measure of residual disease in the primary tumor and in the lymph nodes after neoadjuvant treatment [3,4,5,6]. The categorical RCB classes (0-III) quantify the extent of residual disease, with RCB-0 indicating no residual invasive disease (pCR) and RCB-III indicating a high burden of residual disease [7]. The RCB classes and the RCB calculation on a continuous scale have been validated as a prognostic tool, with a higher RCB after NAC associated with a worse prognosis [8]. In patients undergoing NAC, the ability to identify poorly responding tumors at an early treatment time point can potentially facilitate appropriate early intervention, either by switching to a more effective therapy or by stopping an ineffective but potentially toxic one.

Magnetic resonance imaging (MRI) offers a non-invasive and real-time assessment of the breast cancer treatment response. The functional tumor volume (FTV), derived from dynamic contrast-enhanced (DCE) MRI, is an imaging biomarker that has been integrated into personalized decision-making workflows in the Investigation of Serial Studies to Predict your Therapeutic Response with Imaging and Molecular Analysis 2 (I-SPY 2 TRIAL) to identify and re-direct patients with tumors that respond well or poorly to a more suitable treatment by either de-escalating or escalating the treatment, respectively [9]. However, as a gross measurement of the tumor volume, the FTV does not reflect tumor morphological characteristics such as the shape or structure, which may also predict the response to treatment [10,11]. In BI-RADS, or the Breast Imaging Reporting and Data System, the tumor morphology is described based on observations by radiologists regarding the appearance, shape, and structure in breast imaging, but it is subject to inter-observer variability, especially for non-mass lesions [12]. An objective, quantitative assessment of the tumor morphology in breast DCE-MRI is needed. Previous studies have demonstrated that the tumor sphericity, a single shape feature measured from the same tumor segmentation used in FTV calculation, can predict the pCR for breast cancer treated by NAC [13].

In this study, we extracted a set of morphologic features from the existing FTV tumor segmentation and investigated the additive value of tumor morphologic features to FTV in the prediction of RCB-based pathologic outcomes using data from the multicenter I-SPY 2 study.

## 2. Materials and Methods

### 2.1. Patient Cohort

The study cohort comprised 990 patients enrolled in the I-SPY 2 clinical trial who had completed treatment by November 2016. Women who were 18 years or older and diagnosed with clinical stage II or III breast cancer with a tumor size of at least 2.5 cm by imaging or physical examination were eligible to enroll in the I-SPY 2 TRIAL. Patients who had a hormone receptor (HR)-positive/human epidermal growth factor receptor 2 (HER2)-negative status that were low risk according to MammaPrint (Agendia, Irvine, CA, USA) were excluded. Other patient exclusion criteria included not having a pathologic assessment at surgery or not having demographic data. All patients were treated with experimental drugs or standard chemotherapy for 12 weeks, followed by 4 cycles of doxorubicin and cyclophosphamide (AC). This was a Health Insurance Portability and Accountability Act (HIPAA)-compliant multicenter study. The study was conducted according to the guidelines of the Declaration of Helsinki and approved by the Institutional Review Boards of the participating sites. All participating sites had received institutional review board approval, and all participating patients provided written informed consent for this research. 

### 2.2. Image Acquisition

As per the I-SPY protocol, dynamic contrast-enhanced (DCE) MRI was acquired at multiple time points for each patient at the participating site. MRI examinations were performed on either 1.5T or 3T scanners, but the same scanner and configuration was always used for the same patient across time points. Three-dimensional fat-suppressed T1 images were acquired before and after contrast injection. Details of the DCE-MRI acquisition protocol can be found in Table S1. Postcontrast imaging started simultaneously with the injection. The gadolinium contrast agent was administrated intravenously at a dose of 0.1 mmol/kg body weight and at a rate of 2 mL/s, followed by a 20 mL saline flush. DCE-MRI acquired at pretreatment (T0) and 3 weeks after the start of NAC (T1) was analyzed in this study. The imaging data included in this study can be accessed via the Cancer Imaging Archive (TCIA) [14]. Patients included in this study were required to have MRI at both T0 and T1, with acceptable MRI quality. MRI was excluded if the data were corrupted or the acquisition was not protocol-adherent (across visits) or had severe motion upon visual inspection.

### 2.3. Image Analysis

The MRI variables, including the FTV and tumor morphologic features, were calculated from the DCE-MRI. As described in prior publications [15], the FTV was calculated as the sum of the voxel volumes, in which the voxels were selected automatically using thresholds for the early percent enhancement (PE) and signal enhancement ratio (SER), after a 3D rectangular volume-of-interest box was delineated manually by a site radiologist or a trained imaging coordinator to cover the entire tumor area. The FTV was calculated at each treatment time point, T0 and T1, separately. The ratio of the FTV value at T1 relative to the FTV value at T0 (referred to as FTV Ratio (FTV_R_)) was also calculated to capture the change between treatment time points.

To calculate the tumor morphologic features, three-dimensional shape features were extracted from the existing FTV tumor mask after simple preprocessing steps were applied to the tumor mask to remove noise and ensure that the radiomic features accurately reflected the tumor shape. First, all voxels were resampled to an isotropic voxel size of 1 mm × 1 mm × 1 mm with nearest-neighbor interpolation. Second, a morphological closing operation with a kernel radius of 5 mm was applied to fill small holes. Third, anti-aliasing filtering was applied to the binary image to reduce the aliasing artifacts created by surface generation [16]. The number of iterations and maximum root-mean-square error were 50 and 0.01, respectively. Finally, objects with a size less than or equal to 100 connected voxels were removed. 

**Figure 1:**
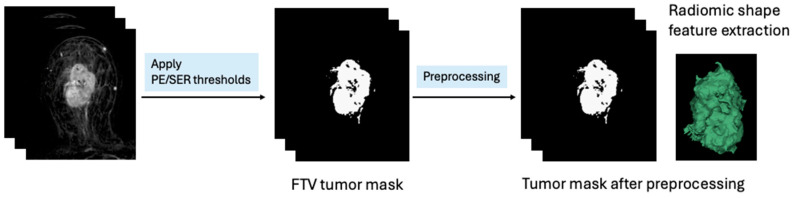
Illustration of radiomic feature extraction from functional tumor volume (FTV) tumor mask in dynamic contrast-enhanced MRI. First, early percent enhancement (PE) and signal enhancement ratio (SER) thresholds were applied to generate the FTV tumor mask, from which the FTV was calculated. Second, multiple preprocessing steps were applied to the FTV tumor mask to fill small holes, smooth edges, and remove small clusters of connected voxels. Lastly, the Pyradiomics package was used to extract radiomic 3D shape features.

FTV tumor masks were generated using in-house software developed in the Interactive Data Language (IDL Version 8.5, NV5 Geospatial Solutions, Broomfield, CO, USA). All preprocessing steps were implemented using the SimpleITK toolkit (Kitware, Inc., Clifton Park, New York, NY, USA) [17]. After preprocessing, radiomic shape features were extracted within the FTV tumor mask. A total number of 17 3D shape features were calculated for the tumor masks in each DCE-MRI exam using Pyradiomics [18]. See Figure 1 for an illustration of the shape feature extraction, and the list of features extracted can be found in Table S2. Missing data occurred when the number of tumor voxels was too small to calculate shape features. In these cases, a random forest-based imputation method was implemented [19]. A total of 51 shape feature variables were extracted, including 17 shape features extracted at T0, 17 shape features extracted at T1, and 17 ratios of shape features at T1 over shape features at T0.

### 2.4. Pathologic Outcome

The pathologic outcome was assessed at the time of surgery, after the completion of NAC. The histopathologic analysis of the surgical specimens was performed at the participating sites by the institutional pathologist, following the I-SPY protocol [6]. RCB classes (RCB-0, RCB-I, RCB-II, RCB-III) were calculated based on pathology measurements [20], where RCB-0 was considered as a pCR. The patient cohort was dichotomized based on the RCB classes in three methods: (1) pCR (RCB-0) versus non-pCR (RCB-I, -II, -III) for the prediction of the best outcome; (2) RCB-0 and -I (RCB-0 and RCB-I versus RCB-II and RCB-III) for the prediction of minimal residual disease; (3) RCB-III versus non RCB-III (RCB-0, -I, -II) for the prediction of the worst outcome with extensive residual disease. This study focused on predicting RCB-III versus non RCB-III. 

### 2.5. Statistical Analysis

The prediction of RCB outcomes was evaluated using four commonly used machine learning (ML) algorithms—elastic net [21], classification and regression tree (CART) [22], random forest [23], and gradient boosting machine (GBM) [24]. Each model was evaluated with respect to its performance using the FTV_R_ and clinicopathologic data, with and without 51 shape feature variables. The patient clinicopathologic data were the age, race, menopausal status, and HR/HER2 subtype defined by HR-positive/-negative and HER2-postive/-negative status. Model evaluation was performed using automatic tuning and testing, in which nested resampling was used. The inner resampling was conduced for the hyperparameter tuning of the model, performed by a grid search using 5-fold cross validation. Lists of the hyperparameters tuned in each ML model can be found in Table S3. The outer resampling was conducted for model generalizability and was performed with 20 stratified subsamples. The area under the receiver operating characteristic curve (AUC) was used to evaluate the predictive performance. AUC estimations were given as the mean with standard deviation (SD) of the AUC values across all 20 testing sets of the outer resampling. An illustration of the nested cross-validation is shown in Figure S1. The model comparison was conducted by the pairwise Wilcoxon signed-rank test. Fisher’s exact test was used to estimate *p*-values for the distributions of categorical clinicopathologic data between the two groups, and a two-sided Student *t* test was used to estimate the *p*-value for the age difference between the two groups. Numeric MRI variables were presented as medians with interquartile ranges, and a two-sided Wilcoxon signed-rank test was used to test the difference in the two groups. Correlation coefficients between the FTV and shape features were calculated by Spearman’s method.

The additive value of the shape features was assessed by comparing the AUCs of the optimal models with and without shape features. The optimal model was selected by identifying the one with the highest mean AUC among the four ML algorithms listed above. Variable importance was used to rank the contributions of MRI and the clinicopathologic variables in the ML models. In the elastic net models, coefficients were used to generate the variable importance for numeric variables only (categorical variables were not included) after the coefficients were scaled by the multiplication of the standard deviation. 

The statistical analysis was performed in R (version 4.2.2). The machine learning model evaluation was performed using the rtemis package [25]. The statistical significance level was nominally set to be 0.05.

## 3. Results

### 3.1. Patient Characteristics

**Figure 2:**
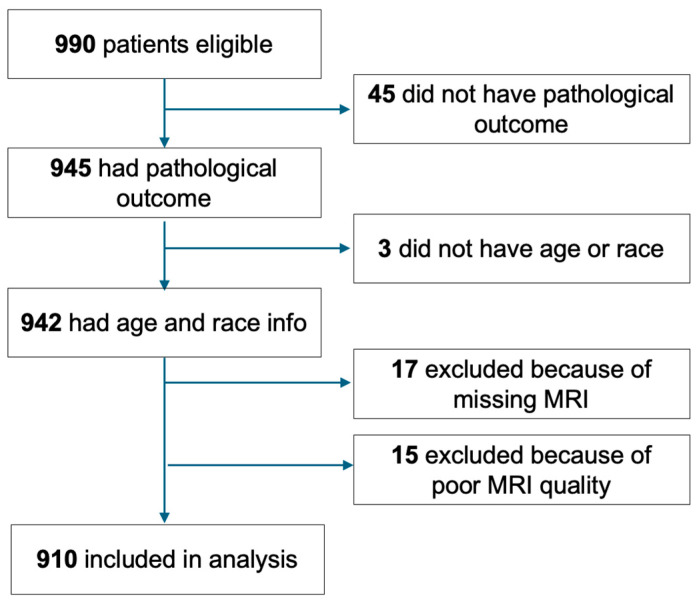
Data inclusion and exclusion. Patients were excluded from the analysis because of missing pathological outcomes, clinicopathologic data, or MRI or unusable imaging data.

A flowchart showing the patient inclusion and exclusion process is shown in Figure 2. Patients with missing pathologic outcomes (n = 45) or clinicopathologic data (n = 3), missing MRI data (n= 17), or poor MRI quality (n = 15) were excluded. Out of 15 excluded due to poor quality, six had non-usable MRI data; six had severe motion; and three had inconsistent sequence acquisition times between T0 and T1 or a non-protocol-adherent sequence acquisition time. Thus, a sub-cohort of 910 was included in the analysis. Table 1 shows the characteristics of the eligible (n = 990), analysis (n = 910), and excluded (n = 80) cohorts. The analysis cohort had the same mean age (48.8 years old) and standard deviation (10.5 years old) as the eligible cohort. The two cohorts also shared similar HR/HER2 and menopausal status, race, and RCB class distributions. However, the excluded cohort had different subtype (*p* = 0.024), race (*p* = 0.018), and RCB class (*p* < 0.001) distributions compared to the analysis cohort.

### 3.2. Additive Value of Shape Features

**Figure 3:**
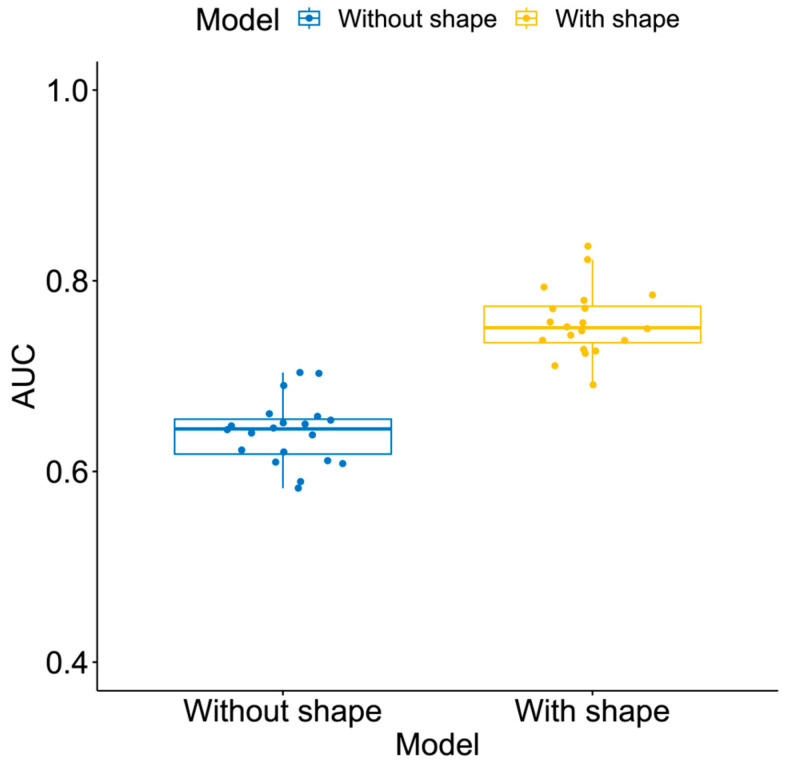
Boxplots of area under the receiver operating characteristic curve (AUC) for prediction of residual disease with and without shape features. AUCs were evaluated by optimal machine learning models independently in 20 stratified subsamples of the analysis cohort (n = 910) for the prediction of RCB-III. Model—without shape: FTV_R_ and clinicopathologic data were used in the predictive model. Model—with shape: shape features were added to the predictive model together with FTV_R_ and clinicopathologic data.

In the analysis cohort, 141 patients (15%) had RCB-III at the surgery and the remaining 769 patients (85%) were non RCB-IIIs (65%). The AUCs of the ML models estimated in the full cohort (n = 910) and in the sub-cohorts defined by HR/HER2 subtype are listed in Table S4. The optimal model for the prediction of RCB-III using the FTV_R_ and clinicopathologic data was based on GBM with an estimated AUC of 0.64 ± 0.03. After the shape feature variables were added together with the FTV_R_ and clinicopathologic data, a higher AUC was achieved (0.76 ± 0.04, *p* < 0.001) by random forest. Boxplots of the AUCs for the models with and without the shape are shown in Figure 3. The AUCs for the prediction of the pCR and RCB-0/-I using the ML models estimated in the full cohort (n = 910) and in the sub-cohorts defined by HR/HER2 subtype are listed in Tables S5 and S6.

**Figure 4:**
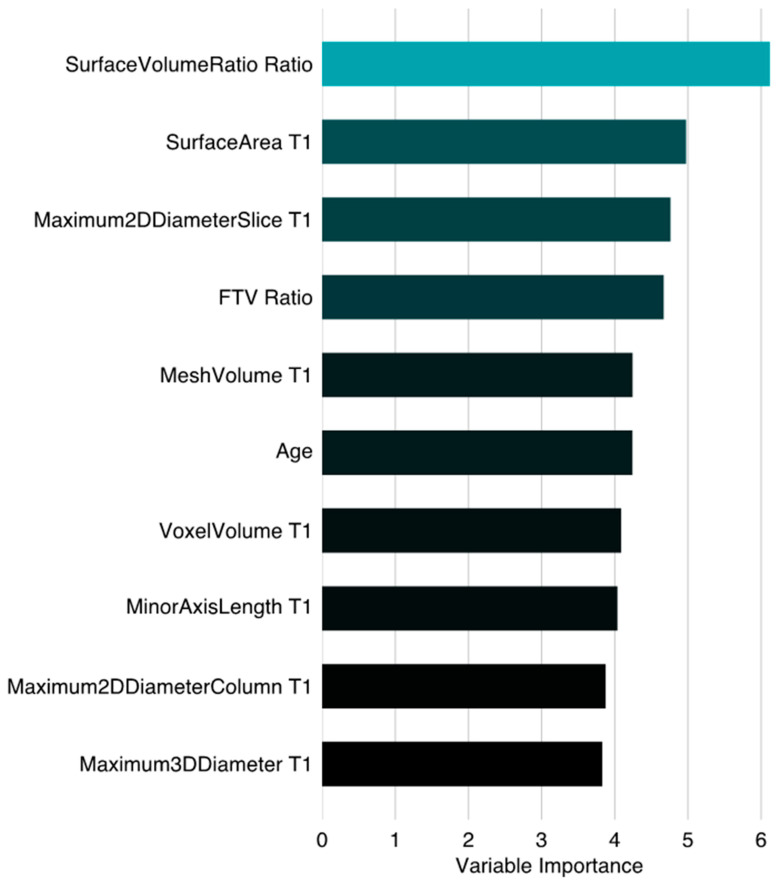
Variable importance for prediction of RCB-III using shape features together with FTV ratio and demographic variables by random forest. Variable importance was ranked according to the mean decrease in accuracy (%) when a variable was excluded. A higher value means that a variable was more important. T0: pretreatment time point. T1: early treatment time point.

**Figure 5:**
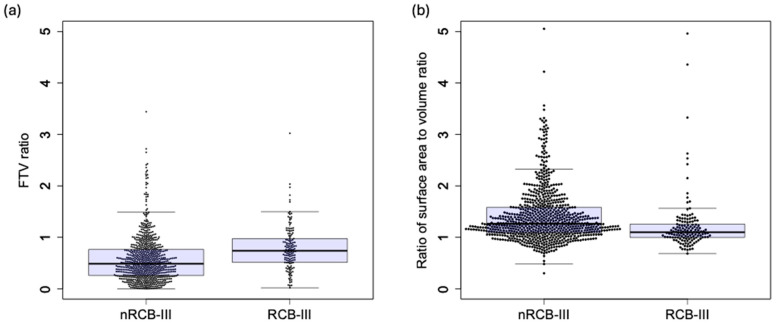
Beeswarm plots with overlaid boxplot of MRI features. (**a**) Functional tumor volume (FTV) ratio between pretreatment and early treatment. (**b**) Ratio of surface area to volume between pretreatment and early treatment. Number of patients in the RCB-III group was 141, with 769 patients in the nRCB-III group (RCB-0, -I, or -II).

The associations between the individual MRI variables (FTV_R_ and shape features) and RCB-III according to a univariate analysis can be found in Figure S3. The Spearman’s correlation coefficients between the FTV and shape features calculated at T0 and T1 are listed in Table S7. The variable importance for the prediction of RCB-III using shape features together with FTV_R_ and clinicopathologic data by random forest is shown in Figure 4, which shows that the most important variable was the ratio of the surface area to volume. The surface area to volume ratio was one of the radiomic shape features analyzed in this study (see Table S2 for the full list), and the ratio represented the change in this shape feature between T0 and T1. The beeswarm plots with overlaid boxplots of the surface area to volume ratio for the RCB-III and non RCB-III groups are shown in Figure 5, which also shows the plots for the FTV_R_. The FTV_R_ was ranked fourth among the top ten contributing variables, and it was significantly higher in the RCB-III group (0.74 [0.51, 0.97]) than in the non-RCB-III group (0.49 [0.26, 0.76]), *p* < 0.001. The surface area to volume ratio was lower in the RCB-III group (1.10 [1.00, 1.26]) than in the non-RCB-III group (1.27 [1.09, 1.58]), *p* < 0.001.

**Figure 6:**
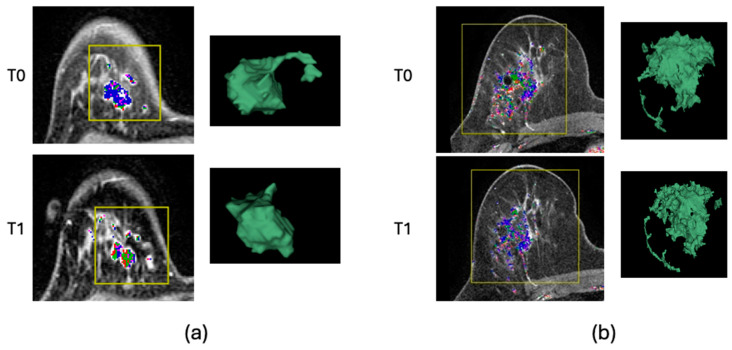
Example cases. Representative slices of post-contrast dynamic contrast-enhanced MRI at pretreatment (T0) and early treatment (T1) time points are shown. Functional tumor volume (FTV) tumor masks were generated by voxels within the region-of-interest box in yellow that had an early percent enhancement (PE) above 70% and are shown superimposed on the representative slices. Colors within FTV tumor masks represent different levels of signal enhancement ratio (SER)—blue: 0 to 0.9; purple: 0.9 to 1.0; green: 1.0 to 1.3; red: 1.3 to 1.75; white: 1.75 and higher. Three-dimensional surface rendering of the preprocessed tumor mask is shown next to the representative slice. (**a**) An example of a patient with RCB-0. (**b**) An example of a patient with RCB-III.

Figure 6a shows an example of the RCB-0 cases. The FTV_R_ at T1 was 0.99, which indicated that the FTV did not change after three weeks of NAC. However, the surface area to volume ratio increased from 0.68 at T0 to 0.87 at T1, resulting in a ratio of 1.28. This example illustrates that, in good responders, the surface area to volume ratio increased even when the FTV remained the same. Figure 6b shows an example of the RCB-III cases. The FTV_R_ was 0.67 but the surface area to volume ratio remained the same (0.38 at T0 and 0.38 at T1). This suggests that, even if the tumor is reduced in size but retains its original shape after three weeks of NAC, it might still indicate a poor response.

### 3.3. Additive Value of Shape Features by HR/HER2 Subtype

**Figure 7:**
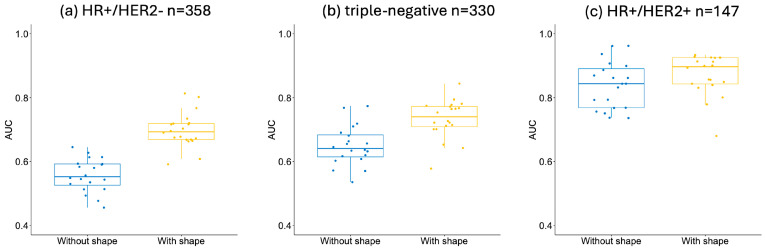
Boxplots of area under the receiver operating characteristic curve (AUC) for prediction of residual disease with and without shape features. AUCs were evaluated by optimal machine learning models independently in 20 stratified subsamples of the analysis cohort (n = 910) for the prediction of RCB-III in (**a**) HR+/HER2−, (**b**) triple-negative, and (**c**) HR+/HER2+. Model—without shape: FTV_R_ and clinicopathologic data were used in the predictive model. Model—with shape: shape features were added to the predictive model together with FTV_R_ and clinicopathologic data.

The same four ML models were built separately in the HR/HER2 sub-cohorts, and boxplots of the AUCs for the models with and without the shape, evaluated in the individual sub-cohorts, are shown in Figure 7. In HR-/HER2+, the number of RCB-IIIs was too low (n = 4, 5%), so the results in this sub-cohort were omitted.

In the HR+/HER2− sub-cohort, the total number of patients was 358, and 81 (23%) of them were estimated as RCB-III at surgery. The optimal model for the prediction of RCB-III using the FTV_R_ and clinicopathologic data was based on the random forest algorithm, with an estimated AUC of 0.56 ± 0.05. After the shape feature variables were added together with the FTV_R_ and clinicopathologic data, a higher AUC was achieved (0.70 ± 0.06) by the random forest, *p* < 0.001.

In the triple-negative sub-cohort, the total number of patients was 330, and 36 (11%) of them were estimated as RCB-III at surgery. The optimal model for the prediction of RCB-III using the FTV_R_ and clinicopathologic data was based on GBM, with an estimated AUC of 0.65 ± 0.06. After the shape feature variables were added together with the FTV_R_ and clinicopathologic data, a higher AUC was achieved (0.73 ± 0.06) by the GBM, *p* < 0.001.

In the HR+/HER2+ sub-cohort, the total number of patients was 147, and 20 (14%) of them were estimated as RCB-III at surgery. The optimal model for the prediction of RCB-III using the FTV_R_ and clinicopathologic data was based on the elastic net, with an estimated AUC of 0.84 ± 0.07. After the shape feature variables were added together with the FTV_R_ and clinicopathologic data, a higher AUC was achieved (0.87 ± 0.06) by the random forest, *p* = 0.073.

## 4. Discussion

This retrospective study leveraged both the imaging and pathological data from the multicenter I-SPY trial and investigated the additive value of radiomic shape features for the FTV from DCE-MRI at pretreatment and after 3 weeks of NAC in the prediction of pathologic outcomes. Our results showed that, when adding radiomic shape features, the AUCs of the predictive models increased in predicting the worst outcome (RCB-III) substantially from 0.64 to 0.76. We also performed the same analysis by breast cancer subtype, defined by HR and HER2 status, and substantial improvements in predicting RCB-III after adding shape features were observed in HR+/HER2- (from 0.56 to 0.70) and triple negatives (from 0.65 to 0.73).

Established in the multicenter I-SPY clinical trial, the FTV is a quantitative imaging marker for the NAC treatment response in women with stage II or III breast cancer [9]. The FTV is currently used as one of the biomarkers in the I-SPY 2 randomization engine to adaptively assign patients to treatment drug arms and to redirect treatment [26,27]. Features that could assist the FTV in predicting pathologic outcomes at early treatment time points would be valuable to improve the performance of MRI-based models. Although the performance of the FTV in predicting the pCR has been reported multiple times over the years [9,28,29,30], the assessment of the FTV in predicting the RCB-III outcome has not been previously discussed. Recently, FTVs measured from 3-week and 6-week MRI have been used to identify inferior responses to NAC by predicting RCB-II/-III in the I-SPY 2 trial [26].

Radiomic analysis is a fast-evolving field of research for radiology and oncology, and radiomic feature extraction is often performed on intensity-based images to quantify the tumor size, shape, texture, and heterogeneity. A large number of image features, including shape features, can be extracted altogether using automated software packages. Associations between radiomic shape features and the treatment response in breast cancer and other types of cancer have been reported previously [31,32,33,34,35]. In a multicenter study of 320 tumors by Granzier et al. [31], a predictive model combining selected pretreatment MRI-based radiomics, including sphericity, and clinical data achieved an AUC of 0.73 in predicting the pCR. Similar shape features (tumor volume, sphericity, and compacity) were found to be predictive of the treatment response to neoadjuvant chemotherapy and tumor recurrence in patients with locally advanced rectal cancer, according to a study by Park et al. [35].

However, the present study was not a typical study that used radiomic features to predict pathologic outcomes. Instead, only radiomic shape features were extracted using existing tumor segmentations generated by FTV calculation, and they were used to quantify the tumor morphology.

This study observed a substantial improvement in predicting RCB-III after shape features were combined with the FTV and demographic data, suggesting that shape features may be associated with drug resistance, especially for HR+/HER2- and triple-negative tumors. Patients with HR+/HER2-, HR+/HER2+, or triple-negative tumors experienced significantly poorer survival when their residual disease was classified as RCB-III compared to those with other RCB classifications, according to a multicenter pooled study of 5161 patients [36]. Therefore, identifying RCB-III early on may aid in the customization of therapeutic strategies to improve patient outcomes. Our results regarding the variable importance demonstrated that most of the top contributing MRI variables were from the early treatment time point, not the pretreatment one. This finding suggests that the tumor morphology or a change in the tumor morphology in DCE-MRI can be more accurate in identifying tumors that are resistant to NAC shortly after treatment initiation compared to before treatment.

While the minimal reduction in tumor size indicated by the FTV could suggest resistance to treatment, our findings indicate that radiomic shape features could offer extra insights into this resistance. The surface area to volume ratio measured between T0 and T1 was ranked as the top contributor based on our variable importance analysis for the prediction of RCB-III, suggesting that non-responding tumors not only maintained their tumor sizes but also tended to maintain their shapes compared to other RCB classes after 3 weeks of NAC. This finding aligns with studies of tumor shrinkage patterns in DCE-MRI during NAC, in which tumor shrinkage patterns were categorized into groups based on concentric shrinkage or non-concentric shrinkage [37,38]. Fukada et al. found statistically significant differences in both the disease-free survival (DFS) and overall survival (OS) between the shrinkage patterns observed in MRI during NAC for low-grade luminal breast cancer [37]. Wang et al. found that the diffuse decrease pattern in HR+/HER2- and stable disease in HER2+ and triple-negative breast cancer could serve as indicators of the response to NAC in their stratified analysis by HR/HER2 subtype [38]. However, both studies were based on the visual assessment of the enhancement patterns on DCE-MRI, which could be subjective.

This study had a few limitations. The data in this study were from a multicenter clinical trial with variability in imaging protocol compliance. A recent study published from I-SPY 2 suggests that protocol compliance and longitudinal variations in FTV segmentation can affect the predictive performance of the FTV [30,39]. Since the tumor masks were generated based on voxels used for FTV calculation, these factors could have affected our analysis as well. Furthermore, our patient population was treated by drug combinations. This treatment heterogeneity could also cause the tumor response to be more heterogeneous, which would make the prediction process more challenging. The current study focused on the relative additive value of radiomic shape features, so we expected a minimal impact on our results. Our cohort was limited by the I-SPY 2 data availability and the targeted drugs used in the trial, so the pathologic outcome rates may be different from the population who receive standard NAC, especially for the best and worst outcomes. However, AUC evaluation is not affected by outcome prevalence, so this limitation should not affect the model’s generalizability. Lastly, this study only included shape features, a small subset of the large number of radiomic features that can be extracted from DCE-MRI, which may limit the additive value of radiomic features. A future study to evaluate the additive value of a comprehensive set of radiomic features with the FTV in predicting the response to NAC is warranted.

## 5. Conclusions

In conclusion, this multicenter retrospective study showed that tumor morphologic features extracted from FTV tumor masks can add value to the FTV in the prediction of RCB-based outcomes early in NAC, particularly for the prediction of RCB-III, the worst residual disease. This improved performance could significantly impact patient care, as it could allow for the timely redirection of patients with poor responses to more effective treatments, thereby increasing their chances of achieving a pCR and improving their prognosis. Future investigation is warranted to assess the tradeoff between false positives and false negatives when the MRI-based model is used in the clinical decision-making for treatment redirection.

## Figures and Tables

**Table 1:** Patient characteristics.

Characteristic	Eligible Cohort n = 990	Analysis Cohort n = 910	Excluded Cohort n = 80	*p*-Value (Analysis versus Excluded)
Age (mean ± standard deviation)	48.8 ± 10.5	48.8 ± 10.5	47.8 ± 11.0	0.41
HR/HER2 subtype (n, %)				0.024
HR+/HER2-	382 (39%)	358 (39%)	24 (30%)	
HR+/HER2+	156 (16%)	147 (16%)	9 (11%)	
HR-/HER2+	89 (9%)	75 (8%)	14 (18%)	
HR-/HER2- (triple-negative)	363 (37%)	330 (36%)	33 (41%)	
Menopausal status (n, %)				0.63
Premenopausal	481 (49%)	438 (48%)	43 (54%)	
Perimenopausal	35 (4%)	33 (4%)	2 (3%)	
Postmenopausal	301 (30%)	282 (31%)	19 (24%)	
Not applicable	135 (14%)	123 (14%)	12 (15%)	
Unknown	38 (4%)	34 (4%)	4 (5%)	
Race (n, %)				0.018
White	783 (79%)	730 (80%)	53 (66%)	
Black or African American	120 (12%)	104 (11%)	16 (20%)	
Asian	68 (7%)	61 (7%)	7 (9%)	
Mixed	7 (0.7%)	7 (0.8%)	0 (0%)	
Native Hawaiian or Pacific Islander	5 (0.5%)	5 (0.5%)	0 (0%)	
American Indian or Alaska Native	4 (0.4%)	3 (0.3%)	1 (1%)	
Unknown	1 (0.1%)	0 (0%)	1 (1%)	
Residual cancer burden (n, %)				<0.001
RCB-0 (pCR)	325 (33%)	315 (35%)	10 (13%)	
RCB-I	135 (14%)	127 (14%)	8 (10%)	
RCB-II	339 (34%)	327 (36%)	12 (15%)	
RCB-III	146 (15%)	141 (15%)	5 (6%)	
Unknown	45 (5%)	0 (0%)	45 (56%)	

HR: hormone receptor. HER2: human epidermal growth factor receptor 2. pCR: pathologic complete response. RCB: residual cancer burden. *p*-values were calculated by two-sided *t* test for age and Fisher’s exact test for HR/HER2, menopausal, and pCR.

## Data Availability

The data presented in this study are openly available in the Cancer Imaging Archive at 10.7937/TCIA.D8Z0-9T85.

## Supplementary Materials

The following supporting information can be downloaded at: https://www.mdpi.com/article/10.3390/tomography10110134/s1, Table S1: I-SPY 2 DCE-MRI acquisition parameters; Table S2: List of 3D shape features extracted using Pyradiomics; Table S3: List of hyperparameters tuned for machine learning algorithms; Table S4: Model evaluation for predicting residual disease defined by RCB-III; Table S5: Model evaluation for predicting pCR; Table S6: Model evaluation for predicting residual disease defined by RCB-II and RCB-III; Table S7: Spearman’s correlation coefficients between FTV and shape features; Figure S1: Illustration of nested cross-validation for machine learning models; Figure S2: Boxplots of area under the receiver operating characteristic curve (AUC) of predicting residual disease with and without shape features; Figure S3: Plots of coefficients and p-values for predicting RCB-III.
