# Supplementary material for: Tumor Morphology for Prediction of Poor Responses Early in Neoadjuvant Chemotherapy for Breast Cancer: A Multicenter Retrospective Study
Source: Tomography. 2024 Nov 20;10(11):1832–45. doi: 10.3390/tomography10110134 (PMC11598075; doi:10.3390/tomography10110134)
Supplement: Supplementary file 1 [file tomography-10-00134-s001.zip › tomography-3270630-supplementary.pdf]

Table S1. I-SPY 2 DCE-MRI acquisition parameters

**Parameter**	**Value**
Sequence type	Gradient echo (GE)
2D or 3D sequence	3D
Slice orientation	Axial
Laterality	Bilateral
Frequency direction	A/P
Phase direction	R/L
FOV - frequency	260 – 360 mm
FOV - phase	260 – 360 mm
Matrix – frequency (acquired)	384 – 512
In-plane resolution	≤ 1.4 mm
Fat-suppression	Active fat-sat recommended
TR	4 – 10 ms
TE	Minimum TE
Flip angle	10 – 20 degrees
B values	N/A
Slice thickness (acquired, not interpolated)	≤ 2.5 mm
Number of slices	≥ 60; complete bilateral coverage
Slice Gap	No gap
Parallel imaging factor	≤ 2
No. of excitations or averages	≤ 2
k-space ordering	-k to +k (standard, non-centric)
Sequence acquisition time	80 sec ≤ scan time ≤ 100 sec
Total post-contrast imaging duration	≥ 8 minutes following injection

Table S2. List of 3D shape features extracted using Pyradiomics

No.	Feature name
1	Mesh Volume
2	Voxel Volume
3	Surface Area
4	Surface Area to Volume Ratio
5	Sphericity
6	Compactness1
7	Compactness2
8	Spherical Disproportion
9	Maximum 3D Diameter
10	Maximum 2D Diameter (Slice)
11	Maximum 2D Diameter (Column)
12	Maximum 2D Diameter (Row)
13	Major Axis Length
14	Minor Axis Length
15	Least Axis Length
16	Elongation
17	Flatness

Table S3. List of hyperparameters tuned for machine learning algorithms

Algorithm	Description	Hyperparameter	values
Random Forest	Many decision trees	Mtry – number of variables randomly sampled as candidates at each split	0.25, 0.5, 0.75, 1 of n.features
Elastic Net	Regularized regression models	Alpha – controls the distribution between LASSO and ridge regression penalties	Seq (0, 1, 0.2)
Classification And Regression Trees (CART)	A binary tree	Prune.cp – penalty to the tree for having too many splits	0, 0.001, 0.01, 0.1
Gradient Boosting	Trees are built sequentially	Shrinkage – learning rate	0.001, 0.01, 0.1, 1

Table S4. Model evaluation for predicting residual disease defined by RCB-III

	Machine learning model	AUC (mean ± SD)	
		Without shape	With shape
Full N = 910, residual rate = 15% (141/910)	Elastic net	0.608 ± 0.057	0.737 ± 0.035
	CART	0.597 ± 0.057	0.636 ± 0.064
	Random forest	0.622 ± 0.034	**0.756 ± 0.036**
	GBM	**0.641 ± 0.033**	0.750 ± 0.033
HR+/HER2- N = 358, residual rate = 23% (81/358)	Elastic net	0.516 ± 0.041	0.672 ± 0.038
	CART	0.526 ± 0.055	0.541 ± 0.055
	Random forest	**0.558 ± 0.051**	**0.699 ± 0.055**
	GBM	0.548 ± 0.054	0.696 ± 0.056
HR+/HER2+ N = 147, residual rate = 14% (20/147)	Elastic net	**0.839 ± 0.073**	0.833 ± 0.079
	CART	0.583 ± 0.103	0.602 ± 0.113
	Random forest	0.707 ± 0.103	**0.871 ± 0.064**
	GBM	0.723 ± 0.103	0.858 ± 0.081
HR-/HER2+ N = 75, residual rate = 5% (4/75)	Elastic net	NA	NA
	CART	**0.489 ± 0.112**	**0.471 ± 0.032**
	Random forest	0.444 ± 0.240	0.463 ± 0.34
	GBM	NA	NA
HR-/HER2- N = 330, residual rate = 11% (36/330)	Elastic net	0.496 ± 0.018	0.702 ± 0.121
	CART	0.560 ± 0.060	0.593 ± 0.071
	Random forest	0.603 ± 0.061	0.717 ± 0.073
	GBM	**0.650 ± 0.062**	**0.733 ± 0.060**

Table S5. Model evaluation for predicting pCR.

	Machine learning model	AUC (mean ± SD)	
		Without shape	With shape
Full<br>N = 910,<br>residual rate = 65% (595/910)	Elastic net	**0.731 ± 0.023**	**0.741 ± 0.028**
	CART	0.668 ± 0.034	0.630 ± 0.033
	Random forest	0.671 ± 0.024	0.671 ± 0.024
	GBM	0.712 ± 0.026	0.715 ± 0.026
HR+/HER2-<br>N = 358,<br>residual rate = 82% (293/358)	Elastic net	**0.625 ± 0.075**	0.574 ± 0.055
	CART	0.515 ± 0.071	0.540 ± 0.053
	Random forest	0.584 ± 0.066	**0.625 ± 0.058**
	GBM	0.606 ± 0.071	0.590 ± 0.070
HR+/HER2+<br>N = 147,<br>residual rate = 61% (89/147)	Elastic net	**0.659 ± 0.062**	**0.649 ± 0.055**
	CART	0.538 ± 0.064	0.545 ± 0.089
	Random forest	0.566 ± 0.062	0.580 ± 0.071
	GBM	0.547 ± 0.067	0.569 ± 0.079
HR-/HER2+<br>N = 75,<br>residual rate = 33% (25/75)	Elastic net	0.484 ± 0.052	0.512 ± 0.079
	CART	0.491 ± 0.104	0.523 ± 0.127
	Random forest	**0.580 ± 0.097**	**0.569 ± 0.132**
	GBM	0.528 ± 0.115	0.482 ± 0.147
HR-/HER2-<br>N = 330,<br>residual rate = 57% (188/330)	Elastic net	**0.640 ± 0.068**	**0.649 ± 0.055**
	CART	0.572 ± 0.055	0.538 ± 0.063
	Random forest	0.562 ± 0.047	0.631 ± 0.052
	GBM	0.607 ± 0.052	0.630 ± 0.042

Table S6. Model evaluation for predicting residual disease defined by RCB-II and RCB-III

	Machine learning model	AUC (mean ± SD)	
		Model without shape	Model with shape
Full N = 910, residual rate = 51% (468/910)	Elastic net	**0.711 ± 0.021**	**0.717 ± 0.028**
	CART	0.662 ± 0.028	0.644 ± 0.034
	Random forest	0.675 ± 0.022	0.692 ± 0.027
	GBM	0.700 ± 0.022	0.715 ± 0.023
HR+/HER2- N = 358, residual rate = 69% (247/358)	Elastic net	0.523 ± 0.045	0.532 ± 0.056
	CART	0.550 ± 0.048	0.538 ± 0.071
	Random forest	0.585 ± 0.041	**0.581 ± 0.045**
	GBM	**0.590 ± 0.038**	0.575 ± 0.054
HR+/HER2+ N = 147, residual rate = 46% (68/147)	Elastic net	**0.730 ± 0.069**	**0.700 ± 0.079**
	CART	0.524 ± 0.091	0.522 ± 0.081
	Random forest	0.650 ± 0.090	0.596 ± 0.083
	GBM	0.630 ± 0.107	0.579 ± 0.096
HR-/HER2+ N = 75, residual rate = 21% (16/75)	Elastic net	0.475 ± 0.051	0.575 ± 0.137
	CART	0.552 ± 0.129	0.571 ± 0.088
	Random forest	**0.653 ± 0.136**	**0.659 ± 0.153**
	GBM	0.610 ± 0.113	0.585 ± 0.164
HR-/HER2- N = 330, residual rate = 42% (137/330)	Elastic net	**0.609 ± 0.079**	0.676 ± 0.061
	CART	0.566 ± 0.055	0.574 ± 0.061
	Random forest	0.573 ± 0.042	0.672 ± 0.053
	GBM	0.607 ± 0.041	**0.683 ± 0.047**

Table S7. Spearman's correlation coefficients between FTV and shape features

Shape feature	T0	T1
Mesh Volume	0.97	0.96
Voxel Volume	0.97	0.96
Surface Area	0.92	0.91
Surface Area to Volume Ratio	-0.75	-0.79
Sphericity	-0.49	-0.39
Compactness1	-0.49	-0.39
Compactness2	-0.49	-0.39
Spherical Disproportion	0.49	0.39
Maximum 3D Diameter	0.84	0.80
Maximum 2D Diameter (Slice)	0.84	0.81
Maximum 2D Diameter (Column)	0.86	0.82
Maximum 2D Diameter (Row)	0.84	0.80
Major Axis Length	0.80	0.72
Minor Axis Length	0.90	0.84
Least Axis Length	0.91	0.89
Elongation	0.15	0.24
Flatness	0.14	0.31

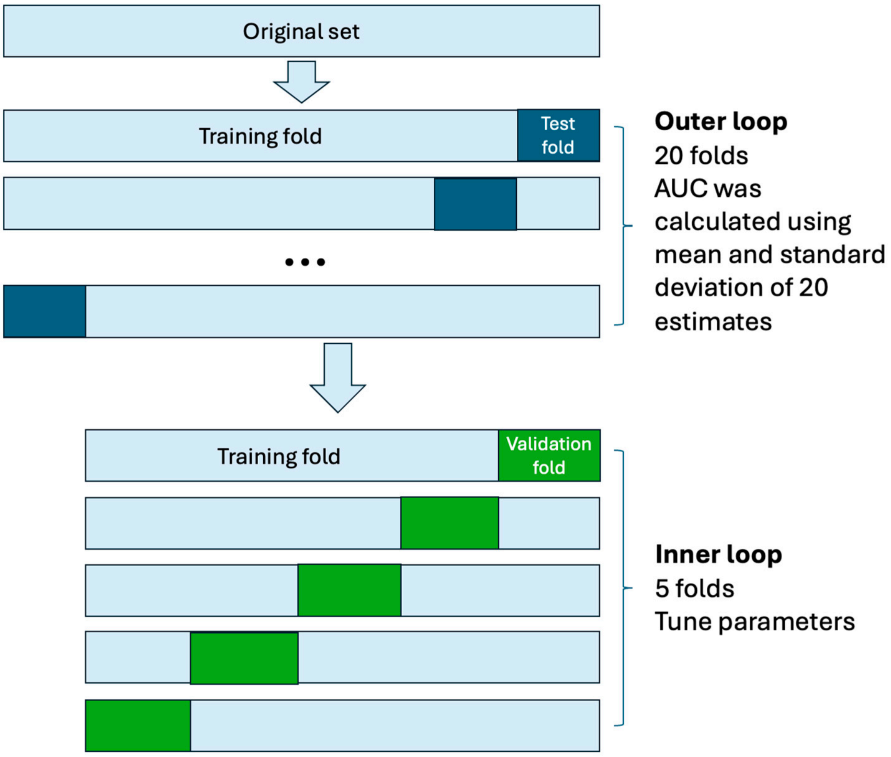

Figure S1. Illustration of nested cross-validation for machine learning models.

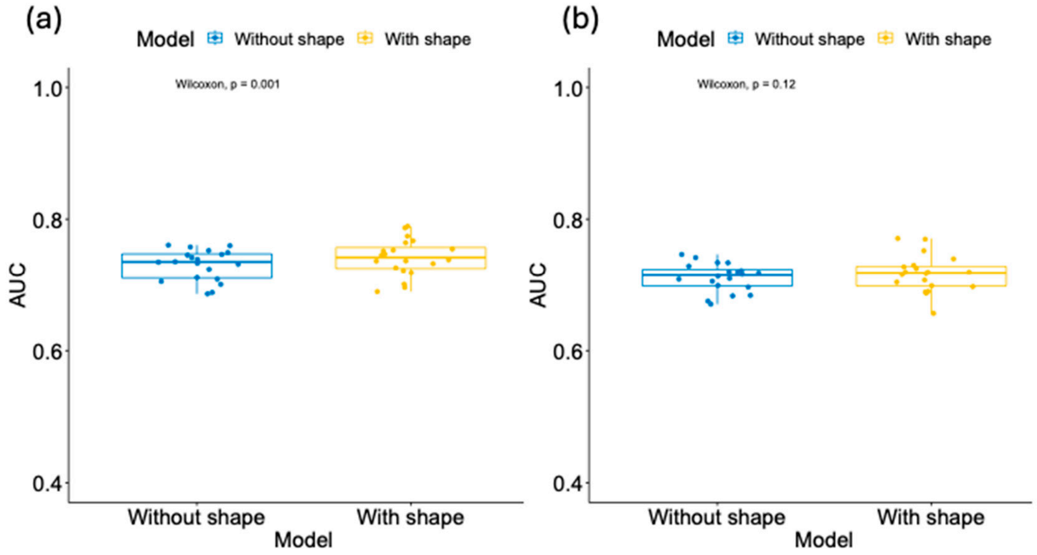

Figure S2. Boxplots of area under the receiver operating characteristic curve (AUC) of predicting residual disease with and without shape features. AUCs were evaluated by optimal machine learning models independently in 20 stratified subsamples of the analysis cohort (n=910) for predicting pCR (a), RCB-0 and RCB-I (b). Model - Without shape: FTV_R and clinicopathologic data were used in the predictive model. Model – With shape: Shape features were added to the predictive model together with FTV_R and clinicopathologic data. The Wilcoxon p-value at the top of each plot was estimated by comparison of 20 AUCs for "Without shape" and "With shape" models. The optimal model for predicting pCR using FTV_R and clinicopathologic data was based on elastic net and the estimated AUC was 0.73 ± 0.02 (mean ± SD). After shape feature variables were added together with FTV_R and clinicopathologic data, higher AUC was achieved (0.74 ± 0.03, p = 0.001) by elastic net. If the analysis cohort was dichotomized by RCB-0, -I (minimum residual disease) versus RCB-II, -III, 442 (49%) patients had RCB-0 or RCB-I and 468 (51%) patients had RCB-II or RCB-III. The optimal model for predicting minimum residual disease using FTV_R and clinicopathologic data was based on elastic net and the estimated AUC was 0.71 ± 0.02 (mean ± SD). After shape feature variables were added together with FTV_R and clinicopathologic data, higher AUC was achieved but did not reach statistical significance (0.72 ± 0.03, p = 0.12) by elastic net.

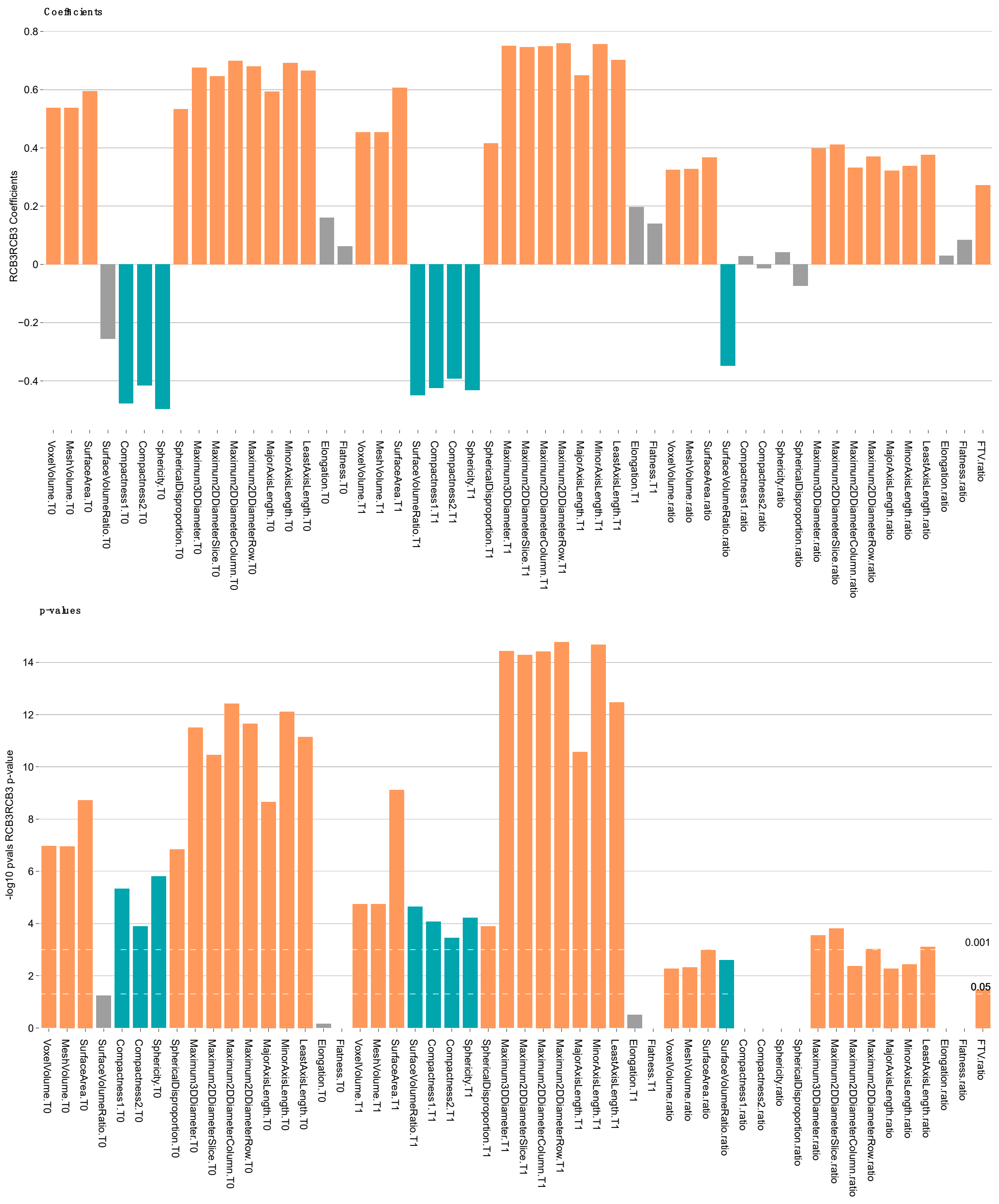

Figure S3. Plots of coefficients and p-values for predicting RCB-III. All variables are plotted with one of three colors: orange represents positive coefficients with $p<0.05$, teal represents negative coefficients with $p<0.05$, gray represents coefficients that are not significant.
